# Polish Refugees in British Lunatic Asylums, 1836–1879: Authority Figures and Social Control Mechanisms

**DOI:** 10.1093/shm/hkaf083

**Published:** 2025-10-31

**Authors:** Emma Harris

**Keywords:** asylum, lunatic, Poor Law, Polish refugee

## Abstract

Drawing on public and organisational records, private correspondence, memoirs, and asylum documentation, this study seeks to explore responses in Britain to mental health problems among Polish refugees from the 1830 November Uprising, and their pathways into British lunatic asylums. After considering their military and refugee experience as a context for mental distress, the article examines ways in which family, community, and above all British and Polish officialdom reacted to mental illness. It suggests that attitudes of middle-class authority figures, initially interacting with mechanisms of the new Poor Law, played a pivotal role in patterns of institutionalisation. These attitudes were based on disdain for the socially and economically disadvantaged, rather than specific ethnic prejudice, while a part was also played by political discrimination, the desire to preserve a favourable public image of the exiles, or linguistic and cultural barriers to communication.

When the Polish refugees of the Great Emigration began to arrive in Britain in noticeable numbers from 1834, the provisions of the new Poor Law provided a changed framework for the institutionalisation of the ‘insane poor’. This study considers the extent of mental health problems among the Polish exiles, taking into account war trauma and the effects of migration. The primary focus is not, however, on reasons for mental illnesses among the refugees, but on the paths of 17 identified lunatics into custodial institutions and the determinants of internment rates. Records of the activities of supervisory British and Polish officials provide insights into mid-century, middle-class attitudes to mental illness and ways in which mechanisms of the Poor Law were employed to institutionalize problematic Poles, contributing to long-standing debate on the use of the new Poor Law to suppress the recalcitrant poor, or more generally to understanding relationships involved in the provision of welfare.^1^ Community and family support are investigated, as well as the impact of political prejudice or linguistic and cultural barriers. Possible differential treatment of Poles on ethnic grounds is explored, with reference to studies of prejudice in the case of nineteenth-century Irish or other minority-group patients.^2^

## The Polish Refugee Community

The term ‘Great Emigration’, used by Polish historians from the mid-twentieth century to designate westward flight following the 1830 November Uprising against Russian rule, suggests a major transnational exodus. In fact, estimates range from 8,000 to 12,000 refugees in all. It was, however, an exceptional expatriation of a regular military force, involving up to a quarter of the defeated troops that crossed the frontier in 1831 to lay down arms in Prussia (28,000) or Austria–Hungary (20,000). Most exiles were officers who accepted sanctuary in France.^3^ Only a small minority reached Britain, almost exclusively those who were unwelcome elsewhere for political reasons. A large contingent came in 1834 when Carbonari-inspired Polish volunteers who had marched to aid democratic movements in Frankfurt and Savoy were expelled from Switzerland, where they had retreated as a unit they named the *Sacred Band* (*Hufiec Święty*). Refused re-admission to France, they moved to London, where they were joined by leading democrats who were banned from both France and Belgium. By June 1834, there were over 200 officer-refugees in Britain, while a roughly equivalent group of other ranks had taken refuge in Portsmouth.^4^ In the late 1830s, the Polish community numbered approximately 600, their refugee status recognized by the award of Government Pensions from June 1834.^5^

Comparable in size to other contemporary communities of European political refugees, the Polish exiles attracted disproportionate attention.^6^ The British press feted their military valour and ‘noble’ origins, combined with suggestions of undeserved social degradation.^7^ British support networks appeared. The longest-lasting was the *Literary Association of the Friends of Poland* [henceforth LAFP], inaugurated in February 1832, inspired by Prince Adam Czartoryski and driven by the enthusiasm of Lord Dudley Coutts Stuart M.P., who was instrumental in pushing through the £10,000 parliamentary grant, which, until October 1838, the LAFP distributed. It continued thereafter to assist the refugees, although its association with conservative *émigré* politics drew increasing criticism from the democratic left wing, and from the mid-1840s, its significance declined.^8^ The Poles also founded their own organisations, which held public meetings and issued proclamations. The General Assembly of the Poles in London (*Ogół Londyński*) aspired to represent the whole community, and societies with specific political aims proliferated.^9^ Even inter-factional discord kept Polish issues in the public eye.

## The Polish Lunatics

No fewer than 17 members of this 600-strong community were confined in British lunatic asylums, and the discussion below is based on their experiences. Key information on these individuals is given in Table 1.^10^ Some were traced through Treasury records of their pension entitlements.^11^ Others were identified through admission books of pauper asylums in the London area, where most of the refugees congregated, and those of the Bethlem Royal Hospital, the Glasgow Royal Lunatic Asylum, and the Hampshire County Lunatic Asylum at Knowle, where Polish private soldiers from Portsmouth were sent after its opening in 1852. It is feasible that there were further cases in provincial asylums. Two of the Polish refugees were already interned in 1836–1837; in 1839, this had risen to four, and by the 1840s, there were six or seven in any one year. Although the sample is small, these figures suggest an elevated incidence of institutionalisation among the Poles. Comparison with rates of internment in the general population is of course problematic, since the Polish community was demographically distinct. There was a striking gender imbalance, with a variant age and marital structure, for they were preponderantly young, single men when they arrived.^12^ Whereas the Lunacy Commissioners’ incidence calculations were based on census data that included minors, at least 40 per cent of the population at the time, the refugees were almost all adults.^13^ Nonetheless, even if the age range is taken into account, the rate of institutionalisation of male Poles seems significantly higher than that for the male population as a whole.^15^ And if the Poles had a higher chance of entering an asylum, they had a lower chance of getting out by any route other than death. While it has been shown that in the mid-nineteenth century up to 60 per cent of all cases were discharged within a year, the rate for the Poles was under 30 per cent (five cases, three of which were rapidly re-interned).^16^

**Table 1.** Members of the Polish Great Emigration admitted to British lunatic asylums from 1836, in order of internment.^14^

Name	Date of birth	Army rank	Date interned	Institution	Date of discharge	Date of death in asylum
1. Jan Stryjkowski U	1811	*2Lt.*	17.05.1836	Hanwell	7.05.1837	—
Source: London Metropolitan Archives (henceforth LMA) H11/HLL/B/03/001, no. 603.						
2. Józef Szulc U	1807	*2Lt.*	2.09.1837	Glasgow RLA	—	30.12.1847
Source: National Health Service, Greater Glasgow and Clyde Archives, Gartnavel Royal Hospital (henceforth NHS, GGCA, GRH) HB/7/15 no. 1091; NHS, GGCA, GRH HB 13/5/28; NHS, GGCA, GRH HB 13/5/23, no. 43; NHS, GGCA, GRH HB 13/6/1, no. 118; NHS, GGCA, GRH HB 13/10/7, no. 1767; NHS, GGCA, GRH HB 13/5/39.						
3. Aleksander Kazimierz Puławski^a^ UG	1800	*chaplain*	21.07.1838	Hanwell	—	29.09.1838
Source: LMA H11/HLL/B/03/001, no. 698.						
4. Karol Czachowski U	1807	*2Lt.*	25.12.1838	Hoxton House	10.01.1839	—
			2.02.1839	Hanwell	—	8.05.1841
Source: LMA H11/HLL/B/03/001, no. 821; LMA H11/HLL/B/16/001, no. 403.						
5. Zygmunt Kossecki U	1807	*2Lt.*	16.03.1838	Bethlem	25.05.1838	—
			21.05.1839	Hanwell	—	29.10.1847
Source: Museum of the Mind (henceforth MM), ARA-19, Series Box Number G6-5; MM, CB-022, 1077; LMA H11/HLL/B/03/001, no. 859; LMA H11/HLL/B/16/001, no. 647.						
6. Tomasz Kraskowski^a^ UG	1803	*2Lt.*	11.12.1839	Hanwell	11.03.1841	—
Source: NA, Home Office (henceforth HO) 27/58. *County of Middlesex. Register of All Persons Charged with Indictable Offences at the Assizes and Sessions Held Within the County in the Year 1839*, 124; LMA H11/HLL/ B/03/001, no. 913; LMA H11/HLL/B/04/001 no. 913; LMA H11/HLL/B/09/001						
7. Benedykt Kolesiński^a^ UG	1807	*Capt.*	Aug. 1843	High Beech	Oct. 1843	—
			12.02.1844	Bethlem	—	20.07.1874
Source: MM, ARA-20 A11/1; MM, ARA-20 Series Box Number G7-5, 15; MM, CB-063 B4-4, no. 47; MM, BAP-046, Series Box Number B7-5; MM, ARA-20, ‘Obituary’, *General Report of the Royal Hospitals of Bridewell and Bethlem,* … *for the Year Ending 31st December, 1874* (London: 1875), 67.						
8. Julia Dydyńska	1799	—	4.03.1844	Hanwell	—	29.10.1871
Source: LMA H11/HLL/B/01/002, no. 1333; LMA H11/HLL/B/19/005, no. 397; LMA H11/HLL/B/05/002 no. 1900.						
9. Piotr Markowski U	1806	*medic.*	19.07.1844	Bethlem	—	12.07.1845
Source: MM, ARA-20, Series Box Number G7-5, 27; MM ARA-20, Series Box Number A11/1; MM, BAP-046, Series Box Number B7-5, no. 152; MM, CB-031, Series Box Number B2-4, 731.						
10. Damazy Łada	1810	*class 2* (+)	2.08.1844	St. Luke's	8.08.1845	—
			13.09.1845	Hanwell	15.05.1847	—
			23.07.1852	St. Luke's	22.10.1852	—
			?	Hanwell	15.05.1877	—
			15.05.1877	Banstead	—	10.01.1879
Source: LMA H11/HLL/B/03/002 no. 1397; LMA H11/HLL/B/04/001 no. 1397; LMA H64/B/05/001/01; LMA H64/B/04/006/01, 123; LMA H64/B/01/015/01, 1; LMA H64/B/01/014, 51; LMA H64/B/04/007, 26; NA Ministry of Health (henceforth MH) 94/24, no. 21699.						
11. Józef Polakowski^b^	1800	*Pte.*	7.05.1845	Hanwell	—	2.05.1846
Source: LMA H11/HLL/B/03/002 no. 1378; LMA H11/HLL/B/04/001, no. 1378,; LMA H11/HLL/B/10/001; LMA H11/HLL/B/06/001 no. 594.						
12. Stanisław Janiszewski	1805	*Lt.*	11.04.1848	Grove Hall	—	26.02.1849
Source: LMA CTHC/01/002; NA MH94/02 no. 292						
13. Michał Warka	1800	*Cpl.*	2.04.1849	Hanwell	—	20.03.1853
Source: LMA H11/HLL/B/04/001, no. 1616; LMA H11/HLL/B/16/001, no. 849; LMA H11/HLL/B/10/001.						
14. Antoni Niemyski	1809	*2Lt.*	25.04.1849	Springfield, Surrey	—	29.04.1851
Source: LMA H46/SP/B/01/109 no. 577; LMA H46/SP/B/05/03.						
15. Marks Drożdżewicz	1800	*Pte.*	10.06.1852	Colney Hatch	—	5.06.1854
Source: LMA H12/CH/B/02/001, no. 616.						
16. Tadeusz Lenkiewicz^a^ U	1804	*2Lt.*	1.06.1853	Hanwell	6.08.1853	—
Source: LMA H11/HLL/B/04/001; LMA H11/HLL/B/20/002, 398; LMA H11/HLL/B/10/001, no. 1904.						
17. Michał Sołdaczuk	1800	*Pte.*	25.01.1860	HCLA Knowle	—	5.02.1860
Source: Hampshire Record Office (henceforth HRO) 48M94-B6-1172b; HRO 48M94-B6-1172c						

U university student before Uprising.

UG university graduate.

(+) civilian refugee granted pension status of officer.

^a^Gold Cross of Valour.

^b^Silver Cross of Valour.

Levels of institutionalisation must be related to specific high-risk factors. The Poles’ army background probably entailed an elevated rate of STD infection and so, potentially, tertiary syphilis and General Paralysis of the Insane [GPI].^17^ More crucially, military service suggests higher rates of mental distress. Duelling and suicide, the latter dubbed a ‘miner's canary’, revealing trauma among veterans, certainly appeared in the community.^18^ The extent of distress, however, defies quantification. While it is tempting to extrapolate from extensive research into the mental-health impact of the American Civil War, Polish veterans’ experiences cannot easily be aligned with these findings.^19^ Separated in time by barely three decades, the wars differed significantly. The Polish-Russian War lasted ten months, with only three major pitched battles.^20^ The particular horrors of warfare were as great, but what Logue and Blanck call ‘the deadly unpredictability of modern wars’ was arguably not yet so apparent, and individual contributions were considered more decisive.^21^ Many American studies focus attention on volunteer or conscripted private soldiers. Meantime, the Polish private soldiers in Britain were mainly from the pre-war regular army, a large contingent from the celebrated fourth regiment of foot.^22^ Half of the *émigré*s in Britain (and three-quarters of the male lunatics) were officers. Many were young volunteers, but older than, for instance, the Confederate soldiers noted by Sommerville to be ‘particularly susceptible to combat anxiety’ because ‘not yet out of their teens’.^23^ Belonging to a generation raised under post-partition foreign rule, they had enthusiastically offered armed resistance to autocracy, internalizing the goal of ‘liberty and independence’.^24^ Their longer-term reactions to warfare might consequently be bracketed with Carroll's judgement that the Civil War had less harrowing mental-health outcomes for black troops.^25^ Nor were the *émigré* veterans subject to the traumatic impact of physical injury, for the wounded rarely expatriated.^26^ And finally, if Carroll's suggestion is accepted, that ‘psychological distress was tied to the war in cases where doctors, families, or the veterans themselves mentioned they believed so’, then there is no evidence that this was true of any of the 17 Poles.^27^

The *émigré* combatants had become ‘refugees’, a status associated with heightened mental health risks, although the word had not yet acquired the connotations of ‘dependency, helplessness and misery’, and association with Post-Traumatic Stress Disorder, that obtained from the second half of the twentieth century.^28^ For the Poles, the dislocating effect of migration on mental health is less clear. Despite experiencing defeat, the Poles’ exile was premised on the idea of victorious return.^29^ They were not usually fleeing death or deportation, for while political leaders faced reprisals, most junior officers could have returned home with few sanctions.^30^ They were buoyed up by a reforming nationalist purpose, apparently demonstrating the quality identified as ‘resilience’.^31^ Unlike Union veterans, they did not have to contend with moral disapproval from their home community.^32^ Instead, they received a heroes’ welcome as they marched West through Germany.^33^ On reaching France, many officers undertook further military education, preparing for the next martial opening. Democrats volunteered for armed expeditions to aid European allies, and others enlisted in the French Foreign Legion, Belgian Army, or Muhammad Ali's Egyptian Army.^34^ In 1848, 1854, and 1863, many exiles again rallied to the challenge of arms.^35^ It is true that they endured culture shock and economic hardship, exhibiting home-sickness, boredom, frustration, and loneliness. Nonetheless, the Poles’ experiences of refugee life offered features that Carroll identifies as strategies to cope with trauma.^36^ The camaraderie of wartime survived in Prussian detention camps, in French refugee *dépôt*s, in internment in Switzerland, in shared households in London, and in the communes in Portsmouth and Jersey. It was fostered in wider community activity, with some turning to religion for comfort, and more turning to politics. It is worth noting that in a study of post-Second-World-War Polish refugees in Britain, the sociologist Jerzy Zubrzycki concluded that those with ‘a sense of security by means of full integration into the Polish Community’ did not exhibit mental health problems, and that it was mainly civilian Displaced Persons who were institutionalised, not military refugees.^37^

Caution should consequently be exercised in assessing the level of trauma-induced incapacity among the *émigré*s. Additionally, certain characteristics of the refugee community indicated a lower likelihood of institutionalisation. Three of the nine conditions specified in 1844 by the Lunacy Commissioners as reasons for asylum internment—congenital idiocy, congenital imbecility and epilepsy^38^—were precluded among the Poles by their primary selection mechanism of military service. A further major category, senile dementia, was ruled out at first by the age group of the refugees. Moreover, their social class and educational attainments, while partly outweighed by the stress of declassing, would have been counter-indicative for internment in the indigenous population.^39^

## Routes into Asylums

The *émigré*s entered institutions through a variety of formal procedures. In London, three men were placed in Bethlem, St. Luke's or a private asylum. Damazy Łada was twice sent to St. Luke's, with fees paid from his government pension; Benedykt Kolesiński was briefly a private patient in Dr Allen's licensed asylum at High Beech, before being interned together with Piotr Markowski in Bethlem, their securities provided by the LAFP.^40^ In Glasgow, Józef Szulc was committed by a magistrate's order under the Scottish system, and his fees were reimbursed from his pension.^41^ The remaining 13 patients entered asylums as pauper lunatics, on two occasions (Puławski, Kraskowski) through the transfer of prisoners found to be insane while serving their sentence.^42^ Eleven patients were therefore committed as paupers within the framework of the Poor Law system.^43^ In the 1830s, the Poles entered the system from domestic situations in proceedings invariably initiated by Anglo-Polish officials via a magistrate's order, which was obtainable with a certificate of lunacy from one doctor, countersigned by a Poor Law officer, although many magistrates also required eyewitness testimony.^44^ It has been demonstrated that the administration of the new Poor Law with regard to pauper lunatics was still evolving through the 1830s, and should be ‘thought of in terms of webs of influence, alliance and distancing between the various persons involved’.^45^ This flexibility can be seen in cases examined below, when officials interested in the incarceration of Polish refugees shopped around parishes and police courts in search of co-operative poor law guardians and magistrates. This was particularly necessary because, with the exception of Kolesiński and Kraskowski who were recently arrived from France at the time of their committal, all the lunatics were on the Government Pension List, and so were not technically paupers.^46^ From the late 1840s, Polish refugees began to acquire the status of pauper lunatics by the standard, indigenous route: three officers and three other ranks were sent to asylums from workhouses.^47^

Only three Poles were admitted to asylums on the basis of potential danger to themselves and/or others, a proportion apparently lower than that in the general asylum population. References to suicide appear in the certification or admission documents of only two of the Poles: Szulc and Sołdaczuk.^48^ Meanwhile, attempted or anticipated suicide has been suggested as a reason for the incarceration of 30 per cent of patients in British asylums in this period.^49^ Violence, or a perceived threat of violence, against others was rarely evidenced against the Poles and was the basis for committal in only two cases. The most conspicuous was that of Tomasz Kraskowski, sentenced in November 1839 for assaulting a waitress in a coffee house in the Haymarket to ten days’ imprisonment, on the expiry of which he was sent to Hanwell.^50^ An attempt to injure a nurse while hospitalized after his attempted suicide was given as an additional reason for the committal of Szulc.^51^ Only three Poles were therefore admitted to asylums on the basis of potential danger to themselves and/or others, none of them from domestic situations.^52^ Overall, there is little indication of the immediate grounds for intervention in committal documentation or admission records, which employed terms such as ‘mental aberration’, ‘acts of eccentricity’, ‘silent and obstinate’, and ‘irritable’.

## The Role of the LAFP

In the first years of London exile, the small Polish community had demonstrated sympathetic attitudes to signs of mental distress, with no inclination towards institutionalisa-tion. In December 1832, when Julian Ursyn Niemcewicz first noticed signs of Czachowski's indisposition, he displayed paternal concern, telling his diary, ‘I don't know how this will develop, but I’m certainly not going to abandon him’.^53^ In 1833–34, Krystyn Lach Szyrma, Secretary of the LAFP, reported the arrival of several ‘poor unfortunate madmen’.^54^ One, the young ensign Marcin Chołod, had in Szyrma's view become mentally ill ‘because of home-sickness; poverty and moral suffering do not improve anyone's health’.^55^ Szyrma approved Dr Józef Szczapiński's proposal that Chołod should be sent to his brother, an *émigré* doctor in Switzerland, who ‘will be the Doctor who can help him most, and the journey itself … may restore him to health’.^56^ At the end of October 1834, Szyrma reported the problems of a more prominent *émigré*, Col. Ludwik Oborski, who had experienced a mental health crisis when commanding the *Sacred Band* in Switzerland.^57^ Oborski believed that his enemies were ‘constantly yapping that he should become a spy …. These voices speak to him day and night. He is so wretched that suspiciousness turns … the most innocent glance into evil’.^58^ Dr. Szczapiński opposed Oborski's planned journey to America, ‘saying that if he went he would lose his bearings altogether’,^59^ but neither Szyrma nor Szczapiński considered institutionalisation when Oborski ignored their advice. After returning precipitately from the United States, he continued to behave erratically and was frequently referred to by the *émigrés* as a ‘madman’.^60^ But someone was always prepared to take him in, and he remained at large, and politically active, throughout his long life in London.^61^ This was, however, increasingly exceptional.

From 1836, the first Polish asylum internments were recorded, almost exclusively initiated in the first decade by LAFP-associated officials.^62^ Their attitudes towards Polish infirmity reveal a desire to control comportment that echoes the approach of middle-class authority figures to indigenous pauper lunatics, categorised by Andrew Scull as ‘the custodial warehousing of … the most difficult and problematic elements of the disreputable poor’.^63^ Functionaries feared the effects of displays of Polish lunacy on British public opinion, and thus on voluntary subscription, or even the Government Grant.^64^ Occasionally, Lord Dudley Stuart was personally involved, but the early internments can be mainly attributed to two self-important, minor officials who appeared in the LAFP in 1836–7.^65^ The foremost of these was John Tebbs, clerk in the War Office, and Treasury-appointed Paymaster to the Polish Refugees until his retirement in 1866.^66^ He worked closely with Dr Edward Straw Blundell, Honorary Secretary to the LAFP from 1837 to spring 1839, and appointed from March 1838 as the LAFP-funded Medical Officer to the Polish refugees.^67^ By June 1839, one *émigré* was writing: ‘Tebbs the Paymaster hostile to us, Blundell the Doctor … hostile to us; the Government which listens to them hostile to us’.^68^ Tebbs was expected to anticipate and report on disruptive behaviour among his charges, which he did with apparent relish, and he was proactive in seeking to remove men from his pay lists for unbecoming behaviour.^69^ Blundell's appearance as Secretary to the LAFP brought the first public mention of mental illness among the refugees; the 1838 Annual Report of the Association noted that ‘the most severe sufferers have, through the instrumentality of the Council, been placed in proper asylums’.^70^

The first recorded pauper internment, at Hanwell in May 1836, was that of Jan Stryjkowski, described as an ‘extremely handsome’ young man’.^71^ The son of a country gentleman, Stryjkowski was a Warsaw medical student who had enlisted as a regimental medical officer in 1830. He arrived in London via Dresden with no resources in November 1833 and suffered considerable privation before the Government Grant was inaugurated in June 1834.^72^ He moved briefly to Belgium, still functioning sufficiently steadily to be elected to the five-man committee of the newly-formed, short-lived *Ogół Polaków* in Brussels.^73^ He returned to London in April 1834 in the company of leaders of the refugee left-wing, Aleksander Kazimierz Puławski and Stanisław Worcell.^74^ Thereafter, he began to demonstrate delusions of grandeur, asserting that descent from the Dukes of Lorraine gave him a better right than Adam Czartoryski to the Polish throne. He wrote to the Princess Victoria and to Nathan Mayer Rothschild, both of which epistles were forwarded to the LAFP. He wrote in French to the Duke of Sussex in March 1836, expressing his ‘desire to repeat to everyone that I deem worthy of my correspondence’ that Poland was being annihilated, and signing himself ‘Legitimate Pretender to form a Dynasty of Kings of the Ancient Poland, Prince Royal’.^75^ Henryk Golejewski's admittedly fanciful autobiography claims that on one occasion, a policeman chased Stryjkowski into the LAFP offices ‘wearing only a shirt and drawers, with wooden clogs, and as he danced around the meeting room one of his clogs broke the glass of a bookcase, and the other broke a window into the street’. Dudley Stuart, says Golejewski, immediately wrote a letter ‘to the lady who admits madmen to Bedlam’, enclosing £5. It is clear that Stryjkowski was never in Bethlem, but in 1836 Mildred, Lady Ellis, was closely involved in running Hanwell, and this detail therefore lends the account authenticity.^76^ Evidence that Dudley Stuart played a role in Stryjkowski's committal can also be found in the LAFP's vote of thanks to ‘Sir William Ellis of the Hanwell Lunatic Asylum’, indicating that the superintendent physician himself had been mobilized by someone with social influence, something also suggested by the two high-ranking magistrates, including one of the Metropolitan Commissioners of Lunacy, who signed the committal papers.^77^ The Hanwell admission record states merely that Stryjkowski's unspecified indisposition had been developing ‘Gradually, for some time past’.^78^

## LAFP Officials and the Poor Law

From 1838, Tebbs and Blundell worked to remove to asylums other men whose behaviour they deemed disruptive, obtaining for their policies the backing of the Lords of the Treasury, responsible for the Polish pension fund. They first collaborated to obtain certification as pauper lunatics of Karol Czachowski and Zymunt Kossecki, junior officers, sons of country gentlemen, law students at the University of Warsaw before the Uprising, who spoke several languages. They were indisputably facing mental health challenges, but they were not deemed a threat to others and did not threaten self-harm. In receipt of government pensions, they were not technically paupers. Kossecki, moreover, lived in a household prepared to look after him. But none of this kept them out of Hanwell. The decision of Tebbs and Blundell to work through the Poor Law system appears unrelated to a belief in effective treatment in public asylums. Their correspondence indicates rather their adverse assessment of the dependent socio-economic status of the Polish refugees. They attached no significance to the fact that they were dealing with officers, ignoring contemporary British army practice that ‘private asylums would provide for officers’, while other ranks were left to ‘the stigmas associated with the pauper asylum’.^79^ At the same time, no particular ethnic prejudice can be detected. In contrast with attitudes to the Irish discussed by Bhavsar and Bhugra in the context of Bethlem, there is no sign that these men viewed the Poles as ‘fundamentally different from their English hosts’ on the basis of ‘differences in religion, … organized sectarianism, and cultural and political differences’, although grounds for these points of disparity could have been exemplified.^80^ They were perhaps influenced by the simpler pauper certification procedures, or even by the financial savings for the Treasury if the parish or County took over the upkeep of refugees with mental illnesses. While Poor Law officials actively sought part-reimbursement of costs from relatives of local paupers, or from foreigners’ wages or assets, the Treasury pension fund made no contribution for the Poles incarcerated at Hanwell.^81^ Tebbs certainly kept a keen eye on the financial implications of institutional-isation in the case of Szulc, whose fees in Glasgow were paid from his pension, pointing out that the cost of his upkeep exceeded the allowance due to a junior officer by £4 p.a.^82^

In order to intern Czachowski and Kossecki as pauper lunatics, Tebbs and Blundell had first to make them chargeable to the parish. Karol Czachowski had arrived in London direct from Prussia in October 1832, and by the end of the year, Niemcewicz records that he was afraid to return at night to his lodgings in Lambeth and then the East End, wandering the streets because he claimed to be followed by spies.^83^ He was nonetheless able to earn his living by teaching painting and by commissions for portraits, while also working on technical inventions, before being awarded a Government Pension in 1834.^84^ In 1838, he was 32 years old, and according to Dr Blundell, of ‘fair complexion, well made, & his bodily health is good’. Blundell had, however, determined by the end of 1838 that Czachowski's mental health necessitated his incarceration, writing to the Lords of the Treasury that his ‘mental aberration’ was expressed in acts of eccentricity, and that he was ‘incapable of managing himself or his affairs’. The only concrete information adduced was that ‘his principal amusement is taking portraits, but which it is needless to add, he never finishes’. Blundell did not mention the persecutory delusions recorded by Niemcewicz, although he was apparently aware of their consequences, since he claimed to have found Czachowski wandering the streets. He did not allege that Czachowski was a danger to himself or others; on the contrary, admitting that ‘He has never been so excited as to render personal restraint necessary but up to the present has always done what he was desired’. Blundell reported that in December 1838, Czachowski's landlord had ‘turned him out of doors’, and on Christmas Day 1838, ‘after much difficulty in procuring the necessary documents, I delivered him up to the Parish Authorities of St. Leonard's Shoreditch, who placed him at Miles's Establishment Hoxton’.^85^ Soon after Christmas, Czachowski discharged himself. Blundell next made an unsuccessful attempt on January 9, 1839 to have him committed to Hanwell through the same parish authorities of St. Leonard's Shoreditch and the magistrates at Worship Street. The *Globe's* court reporter described Czachowski on this occasion as an ‘unfortunate exile’ with ‘a very destitute appearance’, but stressed that ‘his gait and manner showed that he had been in a superior station of life’. His English was apparently good, for he conversed freely with the magistrates, acknowledging a wish to return to his native country, and his replies to their questions ‘were given so pertinently that Mr. Codd and Mr. Broughton said they could not consent to comply with the application’. When the parish surgeon, who was brought in ‘to attest his insanity’, admitted that he had not seen him until the present day, Robert Broughton exclaimed ‘That will not do at all,’ and ‘directed an officer to accompany the unfortunate man to his friends’.^86^

Although the Lords of the Treasury had no statutory powers over lunatics, Blundell now requested ‘instructions’ from them on how to proceed, and presumably received their approval for institutionalisation. He turned to the parish authorities of St Margaret's, Westminster, and also talked to the County Magistrates and the Magistrates of Queen Square, about getting Czachowski into Hanwell.^87^ He renewed efforts to provide evidence of ‘settlement, destitution and of derangement’, which he achieved ‘after some difficulty in furnishing witnesses’, and enquired of the Lords of the Treasury ‘whether the expenses incurred in procuring the witnesses’ could be refunded.^88^ At a hearing at Queen Square on February 2, 1839, Blundell produced Dr A. R. Sutherland, an alienist known to the Queen Square magistrates, to back up the diagnosis of insanity.^89^ When Czachowski told the court that he ‘did not at all consider that he was in a bad state of health, bodily or mentally’, his comrades, who attended the hearing, ‘gave evidence to the contrary’. Nonetheless, the only symptom recorded by the court reporters was connected with his painting: ‘he would paint the portrait of a lady, finish it, deliver it …, then request it back to make some little alteration, and when given back to him, he would put a pair of moustachios on the lady's face, and thus destroy the picture.’^90^ This caught the contemporary journalistic imagination, and a note of the incident, headed ‘A decided case of madness’, was printed in many provincial papers over the following weeks.^91^ The magistrate, W. A. A. White, conducted a long examination, but in the end agreed to send Czachowski to Hanwell, ‘with an order that he should be treated in the kindest manner possible.’ In Blundell's words, Czachowski was ‘safely lodged in the Hanwell Lunatic Asylum the same day’.^92^ No information about him was provided; the admissions register, in contrast with other contemporary entries, contains no note of symptoms. His ‘form of disorder’ was later to be recorded as ‘incoherence’; no other diagnosis survives.^93^ When Blundell inspected him in Hanwell in November 1840, he certified that ‘I find no improvement in his moral condition’.^94^ Six months after this visit, Czachowski died of ‘pulmonary consumption’.^95^

The case of Zygmunt Kossecki illustrates even more clearly the role of British officialdom. Kossecki had experienced mental health problems in France before arriving in London in January 1837.^96^ Despite being awarded a Government pension, he was in trouble by the spring of 1838, when he was admitted to Bethlem on sureties from a hackney carriage driver and a gold lace weaver of Rochester Row, Westminster, in which impoverished district he had been living. It was noted on admission that he was in a ‘destitute condition’, presented a ‘silent and obstinate’ demeanour, and was ‘violent at night’. However, two months later, he was discharged as ‘well’.^97^ At this point, a group of fellow refugees took charge of him. Kossecki had been a member of the *Sacred Band* in Switzerland, and he now went to lodge in Catherine Street, Pimlico, with certain expeditionary comrades, headed by Napoleon Graff and Jan Terlecki, who were trying to make a living as bakers, and who arranged for his pension to be paid directly into their hands for his upkeep. It was an incident connected with this arrangement that put Tebbs on the warpath. He wrote to the Treasury in February 1839: ‘When he presented himself for payment on the 12th Jan. [Kossecki] signed the name of another Refugee instead of his own, since which time, he has become violent and to prevent injury to others I have the honor to submit that instructions may be given to Dr Blundell to place him in the Lunatic Asylum at Hanwell’.^98^ The false signature probably represented a seemingly rational attempt by Kossecki to get an illicit extra payment for himself, since his own pension went to his friends. The manoeuvre may have been repeated for more than one month, as it is recorded on pay day in both October and November 1838 that Kossecki made a voluntary donation of one shilling to the fund set up by the *Ogół Londyński* to support less fortunate brethren.^99^ It was perhaps only in January that Tebbs realized what was happening, and thereafter refused to pay Kossecki's allowance to his comrades.^100^ However, when Blundell approached these comrades to persuade them to make Kossecki homeless, they refused. He wrote to Tebbs:

I find it impossible to comply with the instructions which I recd. from the Lords Commissioners of HMT through you … to place Cs. Sig. Kossecki a Polish refugee of unsound mind in the Hanwell Lunatic Asylum. I have attended three or four times at Queen Square Westminster for a Magistrate's order, but without being able to procure one, Mr White refusing my earnest request, stating as a reason ‘I do not consider Kossecki destitute nor a dangerous Lunatic’.^101^

One such attempt in late February 1839 was reported widely in the press. The *Operative* gave the following details of Kossecki's detention: ‘[O]n the previous evening Dr Blundel (sic) drove up to the station-house, and desired that the prisoner might be kept in safe custody until the morning, when the necessary certificates would be made out for his conveyance to Hanwell Lunatic Asylum. The Doctor further informed the Inspector that … as [Kossecki] was unconscious of his actions it was determined to place him under restraint.’^102^ In court, one of Kossecki's fellow-boarders from Catherine Street gave evidence that ‘his actions had been those of a madman’. He had broken furniture, and went out ‘without his shoes and stockings and cap’, accosting ‘strange gentlemen in the streets’.^103^ The magistrate, the same W. A. A. White who had signed Czachowski's committal a month earlier, pressed the witness for evidence of violent behaviour, and was told that Kossecki had once struck the servant for withholding a razor, but ‘he has never hurt me’. White thereupon declared that Kossecki did not meet the criteria for committal as a pauper lunatic. When Tebbs protested that since the withdrawal of his pension, Kossecki ‘had been living on the charity of his fellow-countrymen’, White pointed out that ‘Your observation does not mend the case’. Kossecki was discharged.^104^ Immediately after this court appearance, Napoleon Graff and Jan Terlecki wrote to the Lords of the Treasury:

Being encumbered with one of our countrymen, Mr Sigismund Kossecki, who is unfortunately deranged, we considered it our duty to take care of him; but as our scanty means did not allow us to provide for existence the Literary Association used to pay his monthly allowance into our hands for which we found him with lodging and board.

Mr Tebbs the present paymaster having declared that he will not pay us his allowance for the future … we have no choice but to abandon him to his fate if your Lordships will not give proper directions to Mr Tebbs for his future support. If his allowance be remitted to us as usual, we are willing to keep him as before.^105^

Tebbs advised the Lords of the Treasury to reject this application ‘in order that the Lunatic may be properly provided for & prevented from committing any outrage upon the public’.^106^ Meanwhile, the search continued for a sympathetic magistrate: ‘Dr Blundell’, wrote Tebbs, ‘has stated the case to Mr Chambers of Bow Street who promised to do what was required in the case but it is absolutely necessary that the Lunatic should first become chargeable to the Parish.’^107^ Tebbs and Blundell finally got their way in May 1839. Kossecki's committal was arranged through the parish of St James, Westminster, with more co-operative magistrates at the Great Marlborough Street police court. He was shipped off to Hanwell, where, like Czachowski, he remained until he died, also from ‘pulmonary consumption’, in 1847. On his death, Hanwell recorded: ‘From Mania to Imbecility’, adding, ‘NB Laboured under Melancholia previous to Admission’—a diagnosis that cannot be found elsewhere in the surviving documentation.^108^

In July 1844, when Tebbs and Blundell took up the case of Damazy Łada—a civilian refugee who arrived in mid-1836 from Galicia via Trieste—they placed him, certified insane by Blundell, in St Luke's, where he was diagnosed with monomania, and stood surety for the payment of his fees from his government pension.^109^ However, a month after his discharge ‘not improved’ in August 1845, Tebbs and Blundell had him committed as a pauper to Hanwell via the Marlborough Street magistrates and the parish authorities of St James, Westminster. From 1844, which was recorded as his ‘first attack’, Łada was in and out of asylums, managing to secure his release on at least one occasion as ‘cured’, before ending his days in 1879 in the newly-opened asylum at Banstead.^110^ His 1844–45 committals constitute the last recorded joint enterprise by Tebbs and Blundell.

## The Role of Karol Szulczewski

Their desire to remove troublesome exiles from public view was duplicated in the 1840s by Polish *émigré* officialdom. This is evidenced in particular by the activities of Maj. Karol Szulczewski, from 1842 Resident Secretary of the LAFP, who orchestrated efforts to incarcerate Benedykt Kolesiński and Piotr Markowski in Bethlem in 1844. Kolesiński, another scion of the landed gentry and former student of the University of Wilno, had fought in a partisan unit before exile in France. Together with his brother Zygmunt, a Wilno-qualified doctor who became a regimental medical officer during the war, he was assigned to *dépôt*s in Besançon and later Caen, before moving to Paris, where he studied at the *École des Mines*.^111^ He had arrived in Britain in unexplained circumstances in the summer of 1843, and was placed almost immediately in Dr Matthew Allen's private asylum at High Beech in Essex.^112^ That this first incarceration was effected by the LAFP is intimated by Szulczewski's involvement, and also by the choice of asylum, for it was here that the only son of Thomas Campbell, the founder of the LAFP, had been resident for nearly fourteen years.^113^ But High Beech was not a prison. In early February 1844, Szulczewski requested that a colleague in France should:

ask around to see whether anyone in Paris knows Captain Benoit de Kolesiński, who has been here for six months. This Lunatic has caused us much trouble and embarrassment. He claims that the Jesuits and the Royal family were persecuting him in Paris and that they’ve followed him here, and so on. He's already escaped from a Madhouse here, and now we’re organizing another hunt for him.^114^

This renewed ‘hunt’ was taking place despite—or was perhaps provoked by—laudatory press accounts of Kolesiński's rescue of a woman drowning in the Serpentine in January 1844. The *Examiner* reported that *‘*The Committee of the Royal Humane Society has addressed a letter to the Literary Association of the Friends of Poland, expressive of thanks to Captain de Kolesinski’.^115^ In mid-February, the ‘hunt’ was over, and the LAFP arranged his admission to Bethlem, with no lesser persons than Lord Dudley Coutts Stuart and Captain John Townshend R.N. (later Marquess Townshend) signing the application and standing security. Szulczewski deposed for the admission records that Kolesiński ‘supposed himself an object of religious persecution, particularly by the Jesuits, who threatened his life’. Equivocating on the question of danger to others, Szulczewski admitted that he had attacked no one, ‘but he is violent’.^116^ After a year in Bethlem, Kolesiński was transferred to the Incurables Division, where he remained for the next 30 years. He considered himself ‘unjustly confined’, and in the 1850s, ‘when a stranger goes through, he … begs his assistance, at the same time giving him a piece of paper with his name on’.^117^

A few months after Kolesiński's confinement, Piotr Markowski joined him in the same hospital, with the same securities. Markowski, who had been a medical student at Wilno before the Uprising, and had enlisted as an army surgeon, was well-known in the London Polish community, reaching England in June 1834, after taking part in the Frankfurt and Savoy expeditions. He must have been seriously discomposed by allegations made against him in 1839 that he was one of seven spies reporting to the Russian Embassy in London, even though the *Ogół Londyński* rejected these findings, and from 1840, appointed him Secretary to its committee.^118^ Earlier on the democratic left of Polish politics, Markowski thereafter moved to the right and remained with the *Ogół Londyński* after its reincarnation in the 1840s as an institution supporting Czartoryski and the LAFP. He continued as Secretary until May 1, 1844, apparently—from the contents of the minutes—functioning efficiently, but already his erratic behaviour was causing concern. In the first week of May 1844, the committee shipped him off to stay with a Polish officer in Scotland for the good of his ‘impaired’ health, but by late June, he was back in London.^119^ Szulczewski now wrote to a friend that he had ‘gone completely crazy’, although the information he provided for Bethlem suggested only eccentricities—that Markowski ‘wishes for an engagement at the Italian Opera, sang three songs in a public place, being a person of very reserved manners. Believes that he has seen visions’. He had not exhibited ‘suicidal leanings’, and on the question of violence towards others, Szulczewski speculated that ‘nothing of the kind has manifested itself but I believe that he might become violent if opposed or allowed liquer (sic)’. In short, he had become an embarrassment in his everyday environment, which included the premises of the LAFP.^120^ Sir Alexander Morison, described as ‘a friend of the Lord [i.e., Dudley Stuart] and of the Poles’, was personally involved in Markowski's confinement, seemingly employing subterfuge in arranging his admission. Szulczewski claimed a cure was soon expected,^121^ but the year that Markowski spent in the hospital brought a rapid decline: ‘When first admitted, he was quiet, orderly, and well conducted. He entertained various delusions …; he consoled himself for what he considered his unjust confinement by the thought of the wealth and power which was in store for him’. He became increasingly weak and died in July 1845 of consumption.^122^

It is conceivable that Kolesiński and Markowski were consigned to Bethlem—perceived as a less degrading institution than Hanwell^123^—because at the time of their institution-alisation they were on the right of the *émigré* political spectrum as Czartoryski supporters aligned with the LAFP.^124^ This placed them in a minority among the Polish lunatics. Most, in keeping with the overall composition of the exiled community in Britain, can be located to the left of the political centre. The most prominent was the unconventional Catholic priest Aleksander Kazimierz Puławski, a leading radical in Warsaw during the Uprising, and one of the five founder-members of the Polish Democratic Association (henceforth TDP) in Paris in 1832. Ten of the sixteen institutionalised male *émigré*s had signed the politically defining 1834 declaration condemning Adam Czartoryski as a traitor to the national cause.^125^ Some had associated in France with radical democrats; several had been members of the *Sacred Band* or of the primitive socialist communes in Portsmouth and Jersey, and occasionally remained active in successor organisations in London.^126^ These views were likely to have further diminished sympathy for their troubles among influential LAFP officials and their associates.^127^

## Attitudes in the Wider *Émigré* Community

Other exiles did not necessarily share the institutionalisation-promoting prejudices and fears of circles associated with the LAFP, but their economic status hampered their ability to offer alternative care. Studies of mid-nineteenth-century asylums have demonstrated the significant role played by the family in committals and length of stay in institutions.^128^ The institutionalisation of Poles can be placed in line with these findings. In the first years, most of the incarcerated exiles had no formal-status family in London, and in only one case did a family in Poland become involved—when the parents of Jan Stryjkowski secured his discharge ‘cured’ from Hanwell in May 1837 and arranged for his return to Warsaw under amnesty.^129^ If attempts were made to activate the homeland families of other patients, they were unsuccessful and went unrecorded. The presence of family did not, of course, constitute a guarantee of domiciliary care. Although Julia Dydyńska had arrived in London as part of an unusually large family unit consisting of a husband and three sons, she was abandoned by them all. Her husband commuted his pension and disappeared to America in the early 1840s.^130^ She stayed briefly in Scotland, as two sons had received scholarships to Glasgow University, where the eldest, Wawrzyniec, achieved distinction as a mathematician and in 1843 graduated as M.D.^131^ None of them questioned Dydyńska's thirty-odd years in confinement, which she spent doing crochet, although she intermittently claimed that her sons had been murdered in Hanwell.^132^

Occasionally, the afflicted could rely on concerned comrades, but generally, community concern was short-term and ineffective. Niemcewicz, whom Czachowski had clearly trusted, and whose social standing might have provided a shield, returned to France a year after his first expressions of solicitude.^133^ Czachowski was not thereafter entirely friendless: albeit not disposed to keep him at home, his comrades and fellow-lodgers attended the court hearing in February 1839 ‘to see him properly disposed of.’^134^ The case of Zygmunt Kossecki confirms the importance of committal decisions of a positively-disposed environment, even if ultimately his household companions had been unable to counteract Tebbs and Blundell. Comradely concern dwindled further as the years passed. Jan Terlecki, active in support for Kossecki in the 1830s, made no apparent move in the 1840s to look after Piotr Markowski, who was then sharing his lodgings in Upper Crown Street, Westminster.^135^ Many of the other Polish lunatics were entirely socially isolated. The ‘exciting cause of the malady’ of Józef Szulc in 1837 was registered in the asylum as ‘Want of Society’.^136^ He was friendless after leaving London to seek employment in Glasgow, although pro-LAFP Glasgow activists and the small local Polish community were involved in his committal, and Felicjan Abdon Wolski, French Master at the High School of Glasgow, stood surety for his fees.^137^ Aleksander Kazimierz Puławski, a few years earlier still a leading authority among the London Poles, had become totally isolated before the events that took him to Hanwell in July 1838. Gossip said that his landlady had evicted him for trying to seduce her daughter. It was rumoured in the summer of 1837 that he had ‘lost his wits’ and was burning his papers, and by November of that year, he was in a debtor's prison until the Poles made a collection to get him out.^138^ But in the bitter winter of 1837–8, he was homeless and wandered the streets at night.^139^ After that, no one in the Polish community knew what had happened to him. He was committed to Hanwell in July 1838 as ‘Antonio Bulaski (a Pole)’, with the information: ‘Insane since his committal to Westminster Bridewell for two months as a rogue and vagabond’. He died in Hanwell two months later.^140^ The *émigré*s knew nothing of his fate; his disappearance was noted, but imaginative rumours, including murder or a return to France, continued to circulate for years.^141^

In general, former comrades showed little interest in asylum inmates after their internment. Ignacy Jackowski, Tebbs’ Deputy Paymaster, visited Hanwell and Bethlem, probably in an official capacity, describing his visits to Czachowski and Kossecki in 1840 as ‘making a votive offering’.^142^ Karol Szulczewski visited Hanwell at intervals from the late 1840s.^143^ In Glasgow, Felicjan Abdon Wolski visited Szulc only once in the asylum, although when informed of his death in 1847, he responded immediately.^144^ A Polish refugee wrote to the asylum from London in 1839, asking how long Szulc would be detained, but there was no further correspondence.^145^ There is only very occasionally evidence of visits to inmates by real friends. Hanwell was situated roughly 15 miles from the districts of London where most Poles lived, and making the trip therefore required considerable resolve; the number of visitors presumably reflected the inmates’ prior community involvement. Michał Warka, at one time a leading member of the Portsmouth commune and politically active thereafter in London, had at least five visitors in the summer of 1849, his first year inside.^146^ On the other hand, there is no record of visits to Bethlem by any of Markowski's numerous associates from his years of activism in the *Ogół Londyński*, even if they turned out to give him a good funeral at Kensal Green—a gesture denied to other asylum inmates, who were generally interred at the institution where they perished.^147^

The only recorded protest about the detention of a Polish patient concerned the private soldier Józef Polakowski, another ex-member of the Portsmouth commune, who had an English wife, and from May 1845 was certified by Blundell and committed to Hanwell. The committee of the *Ogół Londyński* noted at the end of July that his wife, who had visited him several times in Hanwell, claimed he was ‘completely sound of mind’ and could be released if Dr Blundell would sign the requisite document. The committee deprecated this ‘gossip’ and the chairman of the committee himself went out to Hanwell, concluding that Polakowski was still ‘debilitated’. The committee told Polakowski's wife to be more careful in the future with her accusations and ‘wait patiently’ for an improvement in his condition.^148^ There was no improvement: Polakowski died in Hanwell in the autumn of that year of GPI. Tadeusz Lenkiewicz was another asylum inmate with an English wife, who was concerned about him and provided details of his life and condition for the doctors at Hanwell; her role was instrumental in both his committal and his release ‘cured’ in August 1853.^149^

## Linguistic Barriers

Lack of language skills played a demonstrable role in the experience of Polish inmates.^150^ In the 1830s, few of the Polish exiles spoke English. Doctors and asylum staff were therefore usually unable to communicate directly with patients. Józef Szulc's admission notes at the Glasgow asylum stated: ‘The account of this case is but very imperfect, given … the want of the English language in the patient and even in the applicant for his admission’. Later in the month, a doctor recorded: ‘Questioned the patient in French and Latin and when I used the latter he muttered something but so low a tone that I could not make it out’. In March 1838, Szulc ‘speaks readily when questioned but with only imperfect language and so as not to be readily understood’.^151^ In 1841, he was able to express ‘a wish to go home to his father in Poland’, and ‘answer[ed] questions relevantly’. In March 1844, he ‘never speaks or takes notice of anyone except Dr Hutchinson, … with whom he shakes hands and occasionally speaks to in French’. By 1846, Szulc ‘Never speaks or shows any sign of hearing anything said to him except when much annoyed uses a gesture of impatience’.^152^ In Bethlem, Benedykt Kolesiński had similar linguistic problems. As late as 1853, when he had been in the Incurables division for eight years, it was recorded: ‘his delusions regarding the Jesuits still continue accompanied with others which cannot easily be discovered from the very broken English he speaks’. Despite this, the case notes now complained of ‘the coarse and beastly language he uses’; a year later ‘his language is still very filthy’, and in 1873, when he had developed a cancerous tumour which distorted his mouth, ‘he is abusive and foul in language even if harder to understand’.^153^ For illiterate private soldiers, the problem was perhaps worse. In 1860, it was noted on the medical certificate at the committal of Michał Sołdaczuk (admitted as ‘Michael Sowelldonck’) that ‘he talks much to himself with much gesticulation, speaks slowly and with apparent difficulty, but his knowledge of English is so small that I cannot understand his discourse’.^154^

Several of the Poles were described in asylum records as ‘incoherent’, a term that was rarely applied to native speakers of English who were not ‘imbeciles’, and must have been related in part to language skills.^155^ This was recorded of Polakowski, who was diagnosed with GPI, but GPI was not a factor in the case of Czachowski, where the only ‘form of disorder’ surviving in the Hanwell records is ‘Incoherence’.^156^ Nor did GPI figure in the case of Lenkiewicz, who was categorized in the admission register as ‘thin and pale, converses incoherently’.^157^ The last two patients, in fact, had a fair knowledge of English, but linguistic imperfections probably reinforced expectations based on their contemporary economic status. Lenkiewicz, a former teacher of Mathematics at the grammar school in Kowno, received the annotation ‘plain’ in the Hanwell registers to describe his level of education—a term elsewhere applied to clerks, carpenters, and commercial travellers. In this context, it is worth noting that the *Era* court reporter wrote that during Kraskowski's trial, ‘the prisoner went on in an incoherent manner’, while the *Standard* recorded that he was in fact speaking French.^158^

## Cultural Barriers

Two of the Poles were diagnosed in asylum admission and case-book records with melancholia, while six were diagnosed with mania.^159^ This echoes the preponderance of mania found by Bhavsar and Bhugra in diagnoses for nineteenth-century Irish patients in Bethlem, and by Cox, Marland, and York in later-nineteenth-century Lancashire asylums, while in the general asylum population, diagnoses of melancholia predominated. As in the case of other ethnic minorities, the higher level of mania diagnoses can be interpreted as reflecting cultural bias related to physicians’ perceptions of Polish personality traits, or ‘different ways of expressing mental illness’.^160^ The ‘causes of insanity’ recorded for the Poles in admission documentation and case books similarly suggest cultural barriers to effective communication. ‘Political troubles’—which seem to have been rarely discussed in the case of indigenous asylum inmates—were cited in half of the male Polish cases, all officers, where this documentation survives.^161^ The designation may imply a mode of expression that contrasts with the phlegmatic British style, but can be more convincingly related to expectations towards, and preconceptions about, the Polish refugees, underpinned by the narratives of the *émigré* community and British supporters. On the other hand, poverty was mentioned in only one case (Lenkiewicz).^162^ The records provide no sign of recognition that comparative poverty through class displacement—a factor that plausibly influenced the significantly higher institutionalisation of officers—was of any importance.^163^

## Conclusions

This study has demonstrated that a considerable role in hospitalisation rates among mentally ill refugees of the Polish Great Emigration was played by officials associated with the LAFP, whose pro-custodial stance was, in the case of British officials, dictated by a negative assessment of the Poles’ dependent socio-economic status, with political bias perhaps also playing a part. Initially, interacting with Poor Law officials and magistrates, they strove to place distressed exiles in pauper lunatic asylums, ignoring their economic standing as pensioners or community willingness to care for them. This drive towards institutionalisation was, in the 1840s, mirrored by Polish officials of the LAFP, who feared the impact of ‘shameful’ public behaviour. It is therefore feasible that—whatever the incidence of mental illness following the trauma of warfare and the trials of refugee life—the Poles were, like other minorities, liable to higher levels of asylum internment, although there is no indication that they met with the specific cultural discrimination suggested in studies of the Irish. This tentative hypothesis is in some ways reinforced by the fact that the three Polish patients who were discharged ‘cured’ from Hanwell managed in France, Poland, and London to maintain a relatively long-term existence outside institutions. Stryjkowski survived for eight years on his parents’ estate near Warsaw, even if he did not resume his interrupted medical training and died in 1845 at the age of 34.^164^ Kraskowski lingered for a while in London; he then travelled to Spain, before returning disgruntled to France and disappearing from the record in 1849.^165^ Lenkiewicz admittedly gave evidence of confusion and misery later in life, but he also retained a public presence in the activities of the London Polish community throughout the fourteen years that remained to him after his discharge, and was, for example, present at the 1864 meeting in St Martin's Hall that saw the founding of the First International.^166^ The number of Polish lunatics examined in this study is small, but the relatively well-documented background of their lives outside the asylum increases the significance of their experiences.

## Footnotes

1 Jonathan Andrews and Anne Digby, “Introduction: Gender and Class in the Historiography of British and Irish Psychiatry,” in *Sex and Seclusion, Class and Custody: Perspectives on Gender and Class in the History of British and Irish Psychiatry*, eds. Jonathan Andrews and Anne Digby (Leiden: Brill, 2016); Peter Bartlett, “The Asylum and the Poor Law: The Productive Alliance,” in *Insanity, Institutions and Society, 1800–1914*, eds. Bill Forsythe and Joseph Melling (London: Routledge 1999), 57–59; David R. Green, *Pauper Capital: London and the Poor Law, 1790–1870* (London: Routledge, 2010); Alistair Ritch, “Workhouse or Asylum? Accommodating Pauper Lunatics in Nineteenth-Century England,” *Medical History* 67 (2023).

2 Vishal Bhavsar and Dinesh Bhugra, “Bethlem's Irish: Migration and Distress in Nineteenth-Century London,” *History of Psychiatry* 20 (2009); Catherine Cox, Hilary Marland, and Sarah York, “Emaciated, Exhausted, and Excited: The Bodies and Minds of the Irish in Late Nineteenth-Century Lancashire Asylums,” *Journal of Social History* 46 (2012); Claire Deligny, **“**On the Road to the Asylum: Migration and Mental Illness in Victorian Lancashire (c. 1865–1905),” in *International Migrations in the Victorian Era*, ed. Marie Ruiz (Leiden: Brill, 2018); Leonard D. Smith, “Insanity and Ethnicity: Jews in the Mid-Victorian Lunatic Asylum,” *Jewish Culture and History* 1 (1998): 1.

3 Lubomir Gadon, *Emigracya polska: pierwsze lata po upadku powstania listopadowego*, 3 vols (Kraków: Spółka Wydawnicza Polska, 1901), *I,* 22–41; Sławomir Kalembka, *Wielka Emigracja. Polskie wychodźstwo polityczne w latach 1831–1862* (Warszawa: Wiedza Powszechna, 1971), 5–24. In English: Norman Davies, *God's Playground. A History of Poland* (Oxford: Clarendon Press, 1981), 306–33; R. F. Leslie, *Polish Politics and the Revolution of November 1830* (London: Athlone Press, 1956), 256–80.

4 Krzysztof Marchlewicz, *Wielka Emigracja na Wyspach Brytyjskich (1831–1863)* (Poznań: Instytut Historii UAM, 2008), 17–24.

5 The maximum figure, in October 1838, was 625, including 213 private soldiers. The number fell to 390 by April 1844; some had left Britain, others had commuted their pensions and remained. Mieczysław Paszkiewicz, “Lista Emigrantów Polskich otrzymujących zasiłki od Rządu Brytyjskiego w latach 1834–1899. Wstęp,” in *Materiały do Biografii, Genealogii i Heraldyki Polskiej,* eds. Szymon Konarski et al., 10 vols (Buenos Aires: Dorrego, 1964), *II*, 62; Marchlewicz, *Wielka Emigracja*, 25, collates annual numbers, 1831-61.

6 See: Bernard Porter, *The Refugee Question in Mid-Victorian Politics* (Cambridge: CUP, 1979).

7 E.g., *True Sun*, ‘Polish Refugees’, 29 August 1837.

8 Krzysztof Marchlewicz, *Polonofil doskonały. Propolska działalność charytatywna i polityczna lorda Dudleya Couttsa Stuarta, 1803–1854* (Poznań: Wydawnictwo Poznańskie, 2001), 58-61, 180–203, 303–318; Wanda Stummer, “Rola Literackiego Towarzystwa Przyjaciół Polski w kształtowaniu brytyjskiej opinii publicznej w sprawie polskiej (1832–1856),” in *Wielka Emigracja i sprawa polska a Europa (1832–1864),* ed. Sławomir Kalembka (Toruń: Uniwersytet Mikołaja Kopernika, 1980), 45, 61–64.

9 Gadon, *Emigracya Polska*, *III,* 93–99.

10 Six other Poles have been traced; four were of appropriate age to fight in the 1830 insurrection but no conclusive information has been ascertained.

11 Pay lists at National Archives (henceforth NA), Treasury (henceforth T) 1/4099, parts 1 & 2; NA, T50/81–97; NA, Paymaster General's Office (henceforth PMG) 53/2–8; Paszkiewicz, “Lista Emigrantów,” 59–125.

12 There were nine female pensioners.

13 The 1841 census showed almost half the population aged under twenty: *Abstracts of the Answers and Returns made pursuant to Acts 3 & 4 Vict. C. 99, and 4 Vict. C. 7….* (London: HMSO, 1843), xxii. Minors were rarely incarcerated.

14 Primary sources on the asylum history of individuals are listed within the table. Secondary sources providing background on some individuals: Robert Bielecki, *Słownik Biograficzny Oficerów Powstania Listopadowego*, vol 1, (Warszawa: Wydawnictwo Trio, 1995), vols 2–3 (Warszawa: Neriton, 1996–8); Marian Tyrowicz, *Towarzystwo Demokratyczne Polskie 1832–1863. Przewodnik Biobibliograficzny* (Warszawa: Książka i Wiedza, 1964); *Xięga pamiątkowa w 50-letnią rocznicę powstania roku 1830 zawierają*ca *spis imienny dowódzców i sztabs-oficerów, tudzież oficerów, podoficerów i żołnierzy Armii Polskiej w tymż roku Krzyżem Wojskowym “Virtuti Militari” ozdobionych* (Lwów: Drukarnia Ludowa, 1881).

15 The figure for male lunatics institutionalised in England and Wales in 1844 was given by the Commissioners as 12.25 in 10,000; the number in public asylums and licensed houses was 5,521, or 6.9 in 10,000 of the general male population: *Report of the Metropolitan Commissioners in Lunacy to the Lord Chancellor* (London: Metropolitan Commissioners in Lunacy, 1844), 194. For Polish males, the incidence in the 1840s is clearly much higher, reaching c. 100 in 10,000.

16 David Wright, “Getting Out of the Asylum: Understanding the Confinement of the Insane in the Nineteenth Century,” *Social History of Medicine* 1 (1992): 143; David Wright, “The Discharge of Pauper Lunatics from County Asylums in Mid-Victorian England: The Case of Buckinghamshire, 1853–1872,” in *Insanity, Institutions*, eds. Forsythe and Melling, 93; Elaine Murphy, ‘The Administration of Insanity in East London, 1800–1870’ (Ph.D. thesis, University College London, 2000), 195; Andrew Scull, *The Most Solitary of Afflictions. Madness and Society in Britain 1700–1900* (New Haven and London: Yale University Press, 1993), 271.

17 Juliet D. Hurn, “The History of General Paralysis of the Insane in Britain, 1830 to 1950” (Ph. D. thesis, University of London, 1998), 75; Jennifer Wallis, “’Atrophied’, ‘Engorged’, ‘Debauched’: Muscle Wastage, Degenerate Mass and Moral Worth in the General Paralytic Patient,” in *Insanity and the Lunatic Asylum in the Nineteenth Century,* eds. Thomas Knowles and Serena Trowbridge (London: Routledge, 2015), 101.

18 Larry M. Logue, Peter Blanck, *Heavy Laden: Union Veterans, Psychological Illness, and Suicide* (Cambridge: CUP, 2018), 106–107. Polish duelling and suicide are reported, but there has been no systematic study.

19 On the American Civil War: Dillon Carroll, *Invisible Wounds: Mental Illness and Civil War Soldiers*, (Baton Rouge: LSU Press, 2021); Eric T. Dean, *Shook Over Hell: Post-Traumatic Stress. Vietnam, and the Civil War*, (Cambridge Massachusetts: Harvard University Press, 1997); Logue, Blanck, *Heavy Laden*; Diane Miller Sommerville, *Aberration of Mind. Suicide and Suffering in the Civil War–Era South* (Chapel Hill: University of North Carolina Press, 2018).

20 The most comprehensive study of the Polish-Russian war remains: Wacław Tokarz, *Wojna polsko-rosyjska 1830 i 1831* (Warszawa: Volumen, 1993, first published 1928).

21 Logue, Blanck, *Heavy Laden*, 211. In 1830–31, a belief in the individual role in military action is exemplified in insurrectionary memoirs, e.g., Tadeusz Józef Chamski, *Opis krótki lat upłynionych*, ed. Robert Bielecki (Warszawa: PIW, 1989), 124; Henryk Golejewski, *Pamiętnik,* eds. Irena Homoła, Bolesław Łopuszański, and Janina Skowrońska (Kraków: Wydawnictwo Literackie, 1971), 279. On persisting chivalry, e.g., Józef Patelski, *Wspomnienia wojskowe z lat 1823–1831*, ed. Bronisław Gembarzewski (Wilno: Tow. Udz. Kurjer Litewski, 1914), 83, 143; Stanisław Goczałkowski, *Wspomnienia lat ubiegłych*, 2 vols (Kraków: ‘Czas’, 1862), *1,* 151-2; Wiktor Szokalski, *Wspomnienia z przeszłości*, 2 vols (Wilno: Księgarnia Stowarz. Nauczycielstwa Polskiego, 1921), *II*, 39.

22 Carroll, *Invisible Wounds,* 49, notes that soldiers from elite units are less likely to suffer ‘mental wounds’. On the fourth regiment, Józef Święcicki, *Pamiętnik ostatniego dowódcy pułku czwartego piechoty liniowej* (Warszawa: PIW, 1982), 46–47, 246–55. The regiment's mid-century fame can be gauged from W.M. Thackeray's mention in *Pendennis*, 2 vols (London: J. M. Dent & Sons Ltd, 1959), *I*, 217.

23 Sommerville, *Aberration*, 33.

24 Ignacy Radziejowski, *Dziennik powstania 1831* (Warszawa: Wydawnictwo MON, 1973), 74; Chamski, *Opis krótki*, 151-3; Szokalski, *Wspomnienia*, *II*, 6.

25 Carroll, *Invisible Wounds,* 24.

26 e.g., Sommerville, *Aberration*, 44-45. The only Polish patient who had been wounded was Drożdżewicz, although not seriously enough to prevent his return to service: letter from Bartłomiej Beniowski, 3 June 1837, BPP 596, 375, no. 121.

27 Carroll, *Invisible Wounds,* 21.

28 J. B. E. Harrell-Bond and E. Voutira, “Anthropology and the Study of Refugees,” *Anthropology Today* 8 (1992): 7; David Palmer and Kim Ward, “Lost,” *Medicine, Conflict and Survival* 23 (2007): 198; Jolanda De Vries and Guus L. Van Heck, “Quality of Life and Refugees,” *International Journal of Mental Health* 23 (1994): 58–59, 66.

29 Kalembka, *Wielka Emigracja,* 7, 15–16.

30 Ibid., 13–14.

31 Cf Logue, Blanck, *Heavy Laden*, 84; Sommerville, *Aberration*, 235–37.

32 Carroll, *Invisible Wounds,* 167.

33 Gadon, *Emigracya Polska*, *I*, 77.

34 Ibid.*, II*, 251–78, 282, 292–93. 329; *III*, 46.

35 Kalembka, *Wielka Emigracja,* 327 ff., 390, 397–400, 419–432.

36 Carroll, *Invisible Wounds,* 148.

37 Jerzy Zubrzycki, *Polish Immigrants in Britain. A Study of Adjustment* (Dordrecht: Springer-Science+Business Media, BV, 1956) 178,186–87.

38
*Report of the Metropolitan Commissioners*, 102. In 1843, these three conditions accounted for up to 30 per cent of the asylum population.

39 See Table 1: over half of institutionalised Poles were university-educated. Cf. Scull, *Most Solitary*, 353–63; John K. Walton, “Lunacy in the Industrial Revolution: A Study of Asylum Admissions in Lancashire, 1848–50,” *Journal of Social History* 1 (1979): 1.

40 LMA H64/B/05/001/01; LMA H64/B/04/006/01; LMA H64/B/04/007; MM, ARA-20 A11/1; MM, ARA-20/G7-5, 15; MM, ARA-20/G7-5, 27.

41 NHS GGCA GRH HB/7/15, 25.

42 LMA, H11/HLL/B/03/001, no. 698; NA HO 27/58, *Register of All Persons Charged … 1839*, 124; LMA, H11/HLL/ B/03/001, no. 913.

43 Twelve after Łada entered Hanwell in 1845.

44 Murphy, “Administration of Insanity,” 190.

45 Peter Bartlett, **“**The Poor Law of Lunacy: The Administration of Pauper Lunatics in Mid-Nineteenth Century England with special Emphasis on Leicestershire and Rutland” (Ph.D. thesis, University College London, 1993), 147; Peter Bartlett, “The Asylum and the Poor Law. The Productive Alliance,” in Melling and Forsythe, eds. *Insanity, Institutions*, 51.

46 Senior officers received £39 p.a., junior officers £26 p.a.; private soldiers received roughly half; wives received half; after 1848, the officers’ pension was reduced to £17. None of the lunatics commuted their pensions for a lump sum.

47 Janiszewski: Bow; Niemyski: Lambeth; Warka: Mile End; Drożdżewicz: St. Margaret's, Westminster; Lenkiewicz: St. Marylebone; Sołdaczuk: Portsea Island.

48 NHS, GGCA, GRH HB/7/15; HRO, 48M94-B6-1172b; HRO, 48M94-B6-1172c. Michał Warka's disorder was recorded on admission as ‘Mania’, but appeared in the Register of Deaths four years later as ‘Melancholia (Suicidal)’: LMA, H11/HLL/B/04/001, no. 1616; LMA, H11/HLL/B/16/001, no. 849. Cf also Logue, Blanck, *Heavy Laden,* 148.

49 The *General Report of the Royal Hospitals of Bridewell and Bethlem and of the House of Occupations, … for the year ending 31^st^ December 1843* (London: 1844), 42; *Report of the Metropolitan Commissioners*, 102; Anne Shepherd, David Wright, ‘Madness, Suicide and the Victorian Asylum: Attempted Self-Murder in the Age of Non-Restraint’, *Medical History*, 2002 *2,* 175–96, 183, 193.

50 ‘Marlborough Street’, *Standard*, 8 November 1839; ‘Westminster Sessions, November 29^th^’, *Standard*, 30 November 1839.

51 NHS, GGCA, GRH HB/7/15; NHS, GGCA, GRH HB 13/5/28.

52 Bartlett (**‘**Poor Law of Lunacy’, 232) found that in mid-century Leicestershire and Rutland this criterion obtained for over 90 per cent of admissions from domestic situations.

53 Julian Ursyn Niemcewicz, *Pamiętniki. Dziennik pobytu zagranicą. Od Dnia 21 Lipca 1831 r. do 20 Maja 1841*, 2 vols. (Poznań: Księgarnia Jana Konstantego Żupańskiego, 1876-7), *I*, 575. Translations from foreign-language sources are mine.

54 Krystyn Lach Szyrma to Hipolit Błotnicki, 24 June 1833, Biblioteka Czartoryskich (henceforth B. Cz.), MS 5522, 25; Szyrma to Błotnicki, 26 September 1834, B. Cz., MS 5522, 605.

55 Szyrma to Błotnicki, 26 September 1834, B. Cz. MS 5522, 605.

56 Szyrma to Błotnicki, 12 September 1834, B. Cz. MS 5522, 587.

57 Bogusław Cygler, *Pułkownik Ludwik Oborski, szermierz wolności (1789–1873)* (Gdańsk: Wydawnictwo Morskie, 1976), 84–96.

58 Szyrma to Błotnicki, 4 November 1834, B. Cz. MS 5522, 663.

59 Szyrma to Błotnicki, 31 October 1834, B. Cz. MS 5522, 661.

60 Leonard Niedźwiecki to Eustachy Januszkiewicz, 4 December 1838, Leonard Niedźwiecki, *Listy wybrane z lat 1832–1839*, Stanisław Makowski, ed, (Warszawa: Czytelnik, 2008), 446.

61 By 1851, he was living with Jan Kryński and family in Marylebone: NA, HO, 107/ 1488, 17; he was still there in 1871: General Register Office (henceforth RG) 10/ 201/ 94, 27.

62 Only one (Dydyńska) of the committals from domestic situations in this period did not involve the LAFP.

63 Scull, *Most Solitary*, 352.

64 Cf. Akihito Suzuki's ‘shame of public behaviour’: *Madness at Home: The Psychiatrist, the Patient, and the Family in England, 1820-1860* (Berkeley: University of California Press, 2006), 121.

65 Report of the Proceedings of the Fifth Annual General Meeting of the London Literary Association of the Friends of Poland… (London: 1837), 1, 16.

66 NA PMG 53/3, establishment no. 127-128.

67
*Report of the Proceedings of the Seventh Annual General Meeting of the London Literary Association of the Friends of Poland* … (London: 1839), 61. Blundell arrived in London from Hull in the mid-1830s: *Morning Post*, 24 April 1835; *Examiner*, 17 May 1835, and had worked earlier with Tebbs on projects connected with Spanish and Portuguese refugees: *Morning Chronicle*, 5 December 1839.

68 Leonard Niedźwiecki to Piotr Falkenhagen-Zaleski, 11 June 1839, Biblioteka Kornicka (henceforth BK), MS 2413, 1154.

69 See e.g., *The Times*, 17 April 1839, 6; ‘Polish Pamphleteering’, *The Times*, 25 December 1843.

70
*Report of the Proceedings of the Sixth Annual General Meeting of the London Literary Association of the Friends of Poland …* (London: 1838), 24.

71 Golejewski, *Pamiętnik*, *II,* 180.

72 Niemcewicz, *Pamiętniki*, *II,* 173.

73 Idesbald Goddeeris, *La Grande Emigration polonaise en Belgique (1831–1870). Elites et masses en exil à l’époque romantique* (Frankfurt am Main: Peter Lang, 2013), 296.

74 NA HO 5/027, Aliens Entry Book, Port of London 23 April 1834, nos. 955, 956, 959.

75 Letter signed Jean Wladislas Stryjkowski, Londres le 13 mars 1836, B. Cz. MS 5546, 87.

76 Golejewski, *Pamiętnik*, *II,* 180-3.

77
*Report of the Proceedings of the Sixth AGM …*, 16.

78 LMA, H11/HLL/B/03/001, no. 603.

79 Amy Milne-Smith, “Lunacy, Soldiering, and the Abrogation of Care in Nineteenth-Century Britain,” *The Journal of Military History* 88 *(*2024): 450, 658; Howard Ripley, “Military Lunatic Asylum,” *Journal of the Society for Army Historical Research* 83 (2005): 357.

80 Cf. Bhavsar, Bhugra, “Bethlem's Irish,” 185.

81 Murphy, “Administration of Insanity,” 142, 151, 181, 173; Bartlett, “Poor Law of Lunacy,” 57. Most Poles were eventually classified as ‘casuals’ and their costs transferred to the County.

82 J. Tebbs to F. Baring Esq., 11 March 1839; J. Tebbs to F. Baring Esq., 9 July 1839, NA, T 1/4099, part 1.

83 Niemcewicz, *Pamiętniki, I,* 574-5; *II*, 31.

84 Ibid., *I*, 504; *II,* 9, 31, 33.

85 E. Blundell M.D. to F. Baring, M.P., 14 January 1839, NA, T 1/4099, part 1.

86 ‘Worship Street.—Melancholy Case’, *Globe*, 9 January 1839.

87 E. Blundell M.D. to F. Baring, M.P., 14 January 1839, NA, T 1/4099, part 1.

88 J. Tebbs to F. Baring M.P., 14 February 1839, NA, T 1/4099, part 1. The reply was negative.

89 ‘Queen-Square. Assault On A Magistrate’, *English Chronicle and Whitehall Evening Post*, 21 June 1831.

90 ‘Queen Square. The Polish Refugees’, *The News*, 3 February 1839; ‘Queen Square’, *Weekly Chronicle*, 10 February 1839.

91 E.g., *Shrewsbury Chronicle*, 15 February 1839.

92 E. Blundell M.D. to F. Baring M.P., 5 February 1839, NA, T 1/4099, part 1.

93 LMA, H11/HLL/B/03/001, no. 821.

94 Note signed: E. Blundell M.D., 17 November 1840, marked: ‘Recd. 17/11 J.T.’, NA, T 1/4099, part 1.

95 LMA, H11/HLL/B/16/001, no. 403.

96 Bielecki, *Słownik*; Alien Register commencing 1 July 1836, no. 73, NA HO 5/029.

97 MM, ARA-19, G6-5; MM, CB-022, 1077.

98 J. Tebbs to F. Baring, M.P., 20 February 1839, NA, T 1/4099, part 1.

99 Biblioteka Polska w Paryżu (henceforth BPP) MS 602, 1307.

100 ‘A Polish Lunatic’, *Morning Herald*, 28 February 1839: Tebbs stated ‘that the lunatic had received no money since the early part of last month’.

101 E. S. Blundell M.D. to John Tebbs Esq., 27 March 1839, NA, T 1/4099, part 1.

102 ‘Police Intelligence. Queen-Square’, *Operative*, 3 March 1839.

103 ‘A Polish Lunatic’.

104 ‘Police Intelligence. Queen-Square’, *Operative*, 3 March 1839.

105 Napoleon Graff and John Terlecki to Lords of the Treasury (henceforth LT), 1 March 1839, NA, T 1/4099, part 1.

106 J. Tebbs to LT, 4 March 1839, NA, T 1/4099, part 1.

107 Ibid.

108 LMA, H11/HLL/B/03/001, no. 859; LMA, H11/HLL/B/16/001, no. 647.

109 Certificate No. 1044, 4 August 1836, NA, HO 2/8; LMA, H64/B/04/006/01, 123; LMA, H64/B/01/015/01, 1.

110 LMA, H64/B/01/015/01, 1; LMA, H64/B/01/014, 51; LMA, H64/B/04/007, 26.

111 Bielecki, *Słownik*; Józef Alfons Potrykowski, *Tułactwo Polaków we Francji. Dziennik Emigranta*, ed. Anna Owsińska, 2 vols (Kraków: Wydawnictwo Literackie, 1974), *I*, 261-3.

112 MM, ARA-20, A11/1, 111. His brother died in September 1843. Possibly, aware of Benedykt's need for supervision, he arranged that he should seek assistance in London from Dr Wiktor Ratayski, a contemporary medical student in Wilno who had also been in Besançon and Caen.

113 Thomas Campbell, *Literary Reminiscences and Memoirs of Thomas Campbell*, 2 vols (London: Charles J. Skeet, 1860), *I*, 240-2; ‘Commission of Lunacy on the Son of the Late T. Campbell Esq.’, *Scotsman*, 25 September 1844.

114 Karol Szulczewski to Leonard Niedźwiecki, 6 February 1844, BK MS 2409, 158.

115
*Examiner,* 27 January 1844.

116 MM, ARA-20/G7-5, 731.

117 MM, CB-063, B4-4, no. 47.

118
*Polska i Rossiya*, 16, BPP MS 588, 125ff.; President of the General Assembly to Citizen Markowski Piotr [sgd.] Karol Jancewicz, 21 April 1839, BPP MS 594, 491; President of the General Assembly to the London Committee, [sgd.] Karol Jancewicz, 1 May 1839, BPP MS 594, 1037.

119 BPP MS 473; Ignacy Jackowski to Leonard Niedźwiecki, 10 July 1844, BK MS 2405, 662-3; minutes of committee meeting no. 100, 1 May 1844, BPP MS 590, 243; minutes of committee meeting no. 101, 8 May 1844, BPP MS 590, 244.

120 Jackowski to Niedźwiecki, 10 July 1844, BK MS 2405, 662; MM, CB-031, B2-4, 731.

121 Szulczewski to Niedźwiecki, 30 June 1844, BK MS 2409, 185.

122 MM, BAP-046, B7-5, no. 152.

123 Bhavsar, Bhugra, ‘Bethlem's Irish’, 191.

124 Kolesiński supported Czartoryski in 1834, and attempted unsuccessfully to join the French Foreign Legion and the Polish Legion in Spain: Potrykowski, *Tułactwo Polaków*, *I*, 261-3.

125
*Akt z roku 1834 przeciw Adamowi Czartoryskiemu wyobrazicielowi systemu Polskiej Arystokracyi* (Poitiers, 1839), 19, 26, 29.

126 Service Historique de la Défense (henceforth SHD), XL 49, 8. DM, Place d’Avignon, Dépôt des militaires réfugiés polonais, ‘Etat Nominatif des Officiers du Dépôt se qualifiants d’Amis du Peuple, signataires d’un Manifeste Imprimé, qui a troublé la bonne harmonie des Réfugiés’; *Zjednoczenie* 1841, *7*, 10 June 1841, 28.

127 Ignacy Jackowski, the Deputy Paymaster, referred to Puławski as ‘carrion’ and ‘an animal’: Jackowski to Niedźwiecki, 18 February 1841, BK MS 2405, 85.

128 See Scull, *Most Solitary,* 351–6; Wright, ‘The discharge of pauper lunatics’, 104-5.

129
*Report of the Proceedings of the Sixth AGM* …, 24; LMA, H11/HLL/B/03/001, no. 603; ‘Raptularzyk Leonarda Niedźwieckiego’ 1837, 38, BK MS 02415.

130 P. Dydyński to LT, 26 June 1839, NA, T 1/4099, part 1; J. Tebbs to LT, 9 July 1839, NA, T 1/4099, part 1; Adolf Tabasz Krosnowski, *Almanach historique, ou souvenir de l’émigration polonaise* (Paris: 1846), 91.

131
*A Roll of the Graduates of the University of Glasgow from 31st December 1727 to 31st December 1897* (Glasgow: J. MacLehose & Sons, 1898); *Report of the Proceedings of the Seventh AGM* …, 24–5; *Report of the Proceedings of the Eleventh Annual General Meeting of the London Literary Association of the Friends of Poland* (London: 1843), 30.

132 LMA, H11/HLL/B/19/005, no. 397.

133 Niemcewicz, *Pamiętniki*, *I*, 575.

134 ‘Queen Square. The Polish Refugees’, *The News*, 3 February 1839.

135 NA, HO 107/ 738/ 2, 1841, 6.

136 NHS, GGCA, GRH HB 13/5/23, no. 43; NHS, GGCA, GRH HB/7/15, no. 1091.

137 NHS, GGCA, GRH HB 13/6/1, no. 118; *Glasgow Herald*, 29 November 1844.

138 Niedźwiecki to Eustachy Januszkiewicz, 9 November 1837, Niedźwiecki, *Listy wybrane*, 307.

139 Niedźwiecki to Januszkiewicz, 11 January 1838, ibid., 340. On the winter of 1837-38, see Murphy, ‘Administration of Insanity’, 166.

140 Antoni was his baptismal name, Aleksander his religious name and Kazimierz his adopted name. LMA, H11/HLL/B/03/001, no. 698.

141 Niedźwiecki to Karol Sienkiewicz, 31 August 1838, Niedźwiecki, *Listy wybrane*, 386; Niedźwiecki to Eustachy Januszkiewicz, 9 October 1838, ibid., 417.

142 BK MS 2405, 85; MM, VAB-01, F6-3, 8.

143 LMA, H11/HLL/B/20/002, 120.

144 NHS, GGCA, GRH HB 13/5/23, no. 43; NHS, GGCA, GRH HB 13/10/7, no. 1767.

145 Letter from Henryk Kałussowski, NHS, GGCA GRH HB 13/5/23.

146 LMA, H11/HLL/B/32/001.

147 Committee meeting minutes, 17 July 1845, BPP MS 590, 352.

148 Committee meeting minutes, 1 May 1845, 24 July 1845, 31 July 1845, BPP MS 590, 343, 354, 355.

149 LMA, H11/HLL/B/20/002, 398.

150 Compare the situation of Welsh patients: Pamela Michael, David Hirst, ‘The foundation of the North Wales Lunatic Asylum, Denbigh’, in Forsythe, Melling, eds, *Insanity, Institutions*, 167-8.

151 NHS, GGCA, GRH HB 13/5/23, no. 43.

152 NHS, GGCA, GRH HB 13/5/39.

153 MM, CB–063, B4-4, no. 47.

154 HRO 48M94-B6-1172c.

155 Other foreign patients were also described as ‘incoherent’. See e.g., LMA, H11/HLL/B/16/001, no. 898.

156 LMA, H11/HLL/B/04/001, nos. 1378, 1093; LMA, H11/HLL/B/10/001; LMA, H11/HLL/B/16/001, no. 403.

157 LMA, H11/HLL/B/20/002, 398.

158 ‘Marlborough Street’, *Standard*, 8 November 1839; ‘Savage Assault Upon a Female’, *Era*, 10 November 1839.

159 This documentation survives for eleven patients, specifying, sometimes jointly: mania and monomania (six cases), GPI (three cases); dementia (three), melancholia (two), imbecility (one), and incoherence (one).

160 Bhavsar, Bhugra, ‘Bethlem's Irish’, 184, 193-5; Cox, Marland, York, ‘Emaciated, Exhausted’, 512. See also Smith, ‘Insanity and Ethnicity’, 37-39.

161 Walton (‘Lunacy’, 11) cites a case in Manchester; at Hanwell, other foreigners were occasionally accorded political causes, e.g., LMA, H11/HLL/B/16/001, no. 993. On the Poles: LMA. H11/HLL/B/04/001, no. 913; H11/HLL/B/06/001, no. 647; MM, ARA-20 A11/1, 13, 67; LMA, H11/HLL/B/04/001, 1904.

162 Bartlett (‘Poor Law of Lunacy’, 202), found that poverty was listed in Leicestershire and Rutland in 25.3 per cent of cases in patient registers and 11.3 per cent in case books.

163 Until the mid-1840s, there is no record of mental illnesses among other ranks; later, four cases occurred, three supposedly with physical causes: Polakowski (GPI): LMA H11/HLL/B/16/001, no. 849; Warka (‘brain abscesses’): LMA H11/HLL/B/10/001; Drożdżewicz (dementia): LMA H12/CH/B/02/001, no. 616.

164 Konwent Polonia, *Album Polonorum*, vol I, No. A-P 34: http://www.konwentpolonia.pl/album-polonorum/jan-stryjkowski

165 ‘Notes to Correspondents’, *Weekly Dispatch*, 19 December 1841; Thomas Kraskowski to ‘M. Drouin de Thuys, représentant du Peuple, ministre des affaires étrangères’, 10 March 1849, 9 April 1849, AMAE, 38MD/37, 26, 27.

166
*Rocznik Towarzystwa Historyczno-Literackiego w Paryżu*, 1867, 393; Tadeusz Lenkiewicz to Leonard Niedźwiecki, 13 July 1860, BK MS 2406, 554.

